# Epigenetic Inactivation of *Inositol polyphosphate 4-phosphatase B* (*INPP4B*), a Regulator of PI3K/AKT Signaling Pathway in EBV-Associated Nasopharyngeal Carcinoma

**DOI:** 10.1371/journal.pone.0105163

**Published:** 2014-08-15

**Authors:** Jessie Wai-Fong Yuen, Grace Tin-Yun Chung, Samantha Wei-Man Lun, Chartia Ching-Mei Cheung, Ka-Fai To, Kwok-Wai Lo

**Affiliations:** 1 Department of Anatomical and Cellular Pathology, State Key Laboratory in Oncology in South China, The Chinese University of Hong Kong, Hong Kong SAR; 2 Li Ka Shing Institute of Health Science, The Chinese University of Hong Kong, Hong Kong SAR; University of Navarra, Spain

## Abstract

Nasopharyngeal carcinoma (NPC) is a common viral-associated neoplasm in which multiple signaling cascades are interfered with by Epstein-Bar virus (EBV) latent proteins and various genetic alterations. Aside from the previously reported *PIK3CA* amplification, we examined the role of INPP4B, a negative regulator of the PI3K/AKT signaling pathway in the development of NPC. By RT-PCR and Western blotting, we revealed that the expression of *INPP4B* was down-regulated in all five established EBV-positive tumor lines. While *INPP4B* was consistently expressed in normal nasopharyngeal epithelial cells, downregulation of *INPP4B* was found in 32/65 (49.2%) of primary tumors by immunohistochemistry. Furthermore, our study also demonstrated the hypermethylation of the 5′CpG island of *INPP4B* in the tumors in which *INPP4B* transcription was downregulated. Notably, the re-expression of *INPP4B* was detected in the NPC cells treated with the demethylation agent (5-aza-2′deoxycytidine). Our study showed that promoter hypermethylation was the major mechanism for transcriptional silencing of *INPP4B* in NPC. Furthermore, restoration of INPP4B expression significantly suppressed PI3K/AKT downstream signals in the NPC C666-1 cells. *In vivo g*rowth inhibition was clearly demonstrated in the tumor cells stably expressing INPP4B. The findings indicate that epigenetic inactivation of *INPP4B* is one of the key mechanisms in activating PI3K/AKT signaling cascade and playing a role in the tumorigenesis of NPC.

## Introduction

The PI3K/AKT pathway regulates a number of cellular processes, such as cell growth, proliferation, apoptosis, migration, angiogenesis, and glucose metabolism [1]–[3]. PI3K is a major signaling component that transduces signals from various growth factors and cytokines into intracellular messages by generating the second messenger phosphatidylinositol 3,4,5-trisphosphate (Ptdlns(3,4,5)P3) from phosphatidylinositol 4,5-bisphosphate (Ptdlns(4,5)P2) [4]–[5]. This recruits AKT to the plasma membrane where it is subsequently phosphorylated at Threonine 308 and Serine 473 by PDK1 and PDK2 (mTOR2) respectively [6]. Activated AKT translocates to the cytoplasm and nucleus and phosphorylates many downstream targets (e.g. GSK3-beta, FKHRL1, BAD, mTOR, and 4E-BP1) that promote cell proliferation and inhibit apoptosis [1]–[3], [7]–[8]. The tumor suppressor PTEN functions as an antagonist of PI3K [9]. It is a 3-position lipid phosphatase that coverts Ptdlns(3,4,5)P3 back to Ptdlns(4,5)P2 and thus shuts off PI3K/AKT signaling [9]–[10]. Multiple studies have revealed altered expression or mutation of many components (e.g. PIK3CA, PTEN, AKT) of the PI3K/AKT pathway in a broad range of human cancers [1]–[3], [10]–[12]. These genetic and epigenetic changes promote proliferation and survival of tumor cells by activating AKT kinase activity.

Nasopharyngeal carcinoma (NPC) is a distinctive type of head and neck cancer that is closely associated with latent Epstein-Barr (EBV) infection and has a unique pattern of genomic changes [13]. We and others have demonstrated that the regulation of multiple signaling pathways (e.g. NF-kappaB) is disrupted by either viral factors or somatic alterations [14]–[20]. For the PI3K/AKT signaling cascade, Morrison et al (2004) first demonstrated that activated AKT in majority of primary NPC [16]. The constitutive activation of PI3K/AKT signalling pathway may contribute to cell proliferation, survival, migration and genomic instability of this epithelial cancer. In the NPC cases with LMP1 or LMP2A expression, the PI3K/AKT signalling pathway is thought to be activated by these viral latent proteins [17]–[18], [21]. Notably, amplification and mutation of PIK3CA were also reported in 10–20% of this EBV-associated epithelial cancer [22]–[23]. The findings suggest that both viral oncoproteins and genetic alterations contribute to the dysregulation of PI3K/AKT signaling. Recently, Gewinner *et al*. showed that inositol polyphosphate 4-phosphatase type II (INPP4B) plays a critical role in suppressing PI3K/AKT pathway [5]. INPP4B hydrolyzes phosphatidylinositol-3,4-bisphosphate (Ptdlns(3,4)P_2_) which directs AKT plasma membrane engagement. Thus, the loss of INPP4B expression in human epithelial cells leads to constitutive association of AKT-PH domain with the plasma membrane, increased AKT activation and enhances tumor formation [4]–[5]. In this study, we explored whether inactivation of *INPP4B* play a critical role in activation of this oncogenic signaling pathway in NPC. Our findings demonstrated that *INPP4B* was frequently silenced in NPC via promoter methylation and its inactivation contributed to activated PI3K/AKT signaling in this EBV-associated cancer.

## Materials and Methods

All primary NPC specimens were recruited from Department of Anatomical and Cellular Pathology at Prince of Wales Hospital with patients' written consent. The study protocol was approved by the Joint CUHK-NTE Clinical Research Ethics Committee, Hong Kong (CREC Ref. No. 2010.102). The procedures for in vivo tumorigenicity assay in nude mice were approved by the Animal Experimentation Ethics Committee (AEEC) of The Chinese University of Hong Kong, Hong Kong SAR.

### Cell lines, xenografts and primary tumors

NPC cell lines (C666-1 and HK1) and xenografts (C15, C17, xeno-1915 and xeno-99186) were included in this study and maintained as described previously [19], [24]-[25]. A SV40 large T-immortalized normal nasopharyngeal epithelial cell line NP69 was used as control [26]. For immunohistochemistry (IHC) study, a total of 65 primary NPC patients were recruited with written consent and their archival formalin-fixed paraffin-embedded tumor specimens were collected from the tissue bank of the Department of Anatomical and Cellular Pathology at Prince of Wales Hospital. The study protocol was approved by the Joint CUHK-NTE Clinical Research Ethics Committee, Hong Kong. All specimens were taken before treatment and were histologically evaluated to be EBV-positive undifferentiated or poorly differentiated carcinomas as demonstrated by EBER in-situ hybridization. The clinical parameters are listed in Table 1.

**Table 1:** Characteristics of the EBV-positive NPC Patients (n = 65).

Sex (No. of Male/Female)	50/15
Age (Yrs, Mean ± SE)	50.42±1.29
Clinical Stage	No. of patients
1	7
2	17
3	22
4	19
Disease-free Survival Time (Months, Mean ±SE)	36.19±2.12
Total Score of INPP4B expression	% of patients
Absence/Weak (Score = 0–3)	49.32
Intermediate/High (Score>3)	50.77

### RT-PCR

The transcription of *INPP4B* was determined by RT-PCR analysis as described previously [27]. Expression of β*-actin* was used as a control for each sample. The primers used are listed in Table S1.

### Western blotting and IHC analysis

Expression or phosphorylation of INPP4B, AKT, PTEN, mTOR, GSK3α/βand ACTIN in the tumor samples were determined by Western blotting. The primary antibodies used are shown in the Table S2. The intensities of protein expression were quantified by densitometric scanning using ImageJ software. The expression of INPP4B was also assessed in 65 paraffin-embedded primary tumors using a semi-quantitative method [19]. The INPP4B expressing cells were counted and scored according to their prevalence and intensity among the tumor cells. The INPP4B expression score was the product of proportion and intensity scores, ranging from 0 to 12. The INPP4B expression was categorized into absence (score 0), low (score 1–3), intermediate (score 4–6), and high (score 7–12) and then correlated with respective clinical parameters.

### Bisulfite sequencing and methylation specific PCR (MSP)

Bisulfite sequencing and MSP were performed as described previously [28]. The DNA samples were subjected to bisulfite modification using the EZ DNA Methylation-Gold Kit (Zymo Research). A total of 74 CpG sites spanning approximately 717-bp on the 5'CpG island of *INPP4B* were analyzed by bisulfite sequencing. This region covered the critical transcriptional regulatory domains sufficient for *INPP4B* expression in epithelial cells [29]. The PCR primers for bisulfite sequencing and MSP assay are listed in Table S1.

### Restoration of INPP4B expression in C666-1 cells

Full length *INPP4B* was amplified from a TrueORF cDNA clone (Origene, Rockville, MD) and inserted into pCMV6 (OriGene) to produce the *INPP4B* expression vector. pCMV6-*INPP4B* and the vector only were transfected into C666-1 cells using LipofectAMINE 2000 (Invitrogen, Carlsbad, CA, USA) as described previously [28]. The restoration of INPP4B expression was confirmed by Western blot analysis.

### Cell proliferation and *in vivo* tumorigenicity assays

The cell viability and proliferation of the *INPP4B*-expressing C666 cells was detected by WST-1 assays (Roche) [19]. For the *in vivo* tumorigenicity assay, four nude mice (6 to 8 week old) were injected subcutaneously with 2×10^6^ INPP4B-expressing or control C666-1 cells. Subcutaneous tumor growth was monitored for 28 days by caliper measurements of the tumor size [28].

## Results

### Downregulation of *INPP4B* in NPC

**Figure 1:**
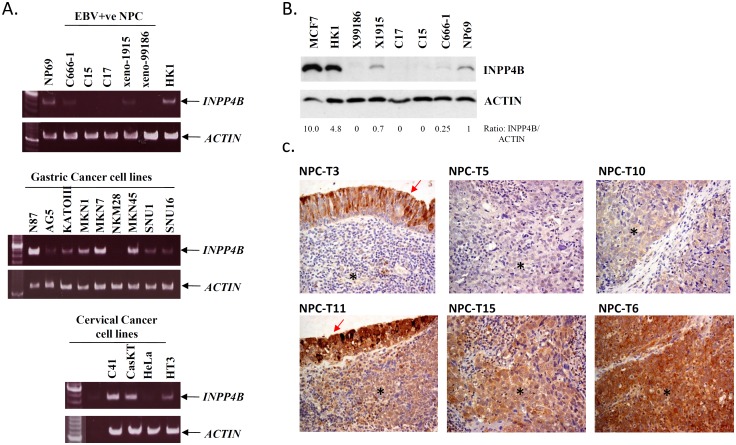
Expression of INPP4B in NPC. (A) RT-PCR analysis of *INPP4B* transcription in NPC, gastric and cervical cancer cell lines. The reduced expression of *INPP4B* was detected in the EBV+ve NPC tumor lines when compared with the EBV-negative NPC cell line HK1 and immortalized nasopharyngeal epithelial cells NP69. Loss of *INPP4B* transcription was also detected in a gastric cancer cell line NKM28 and a cervical cancer cell line HeLa. The RT-PCR experiments were performed in duplicate. (B) The reduced INPP4B protein expression of EBV-positive NPC tumor lines was detected by Western blotting. A high level of INPP4B expression was found in the EBV-negative NPC cell line HK1. The experiment were carried out in duplicate (C) By IHC staining, a reduction in, or loss of, INPP4B expression was detected in the NPC tumor T3, T5 and T10. In tumor T3, intensive staining of INPP4B was demonstrated in the normal nasopharyngeal epithelial cells (red arrow). Representative NPC cases with medium (T11 and T15) and high expression levels of INPP4B (T6) are shown. NPC tumor cells are indicated (*).

To assess the role of *INPP4B* in the activation of PI3K/AKT pathway in NPC, we conducted RT-PCR and Western blotting to detect its expression in a panel of NPC cell lines and patient derived xenografts (PDXs). As shown in Figure 1A, the transcription of *INPP4B* was greatly reduced or completely lost in five tumor lines, while its expression was detected in the immortalized nasopharyngeal epithelial cells NP69 and an EBV-ve NPC cell line, HK-1. A similar INPP4B protein expression pattern was also detected in these samples by Western blotting (Figure 1B). In addition to NPC, we also examined the *INPP4B* expression in other epithelial cancers. However, the downregulation of *INPP4B* was only found in 1/9 (11.1%) gastric cancer cell lines and 1/4 (25%) cervical cancer cell lines. This finding implies that the inactivation of *INPP4B* is a common event in EBV-associated NPC. Using immunohistochemistry, we further assessed the INPP4B expression in the primary tumors. INPP4B was consistently expressed in the normal nasopharyngeal epithelia. Reduction or loss of INPP4B expression (score 0–3) was found in 32/65 (49.2%) of primary tumors (Figure 1C). As shown in Table 2 and Figure S1, INPP4B expression was not correlated with staging and clinical outcome of the patients.

**Table 2:** Correlation between INPP4B expression and clinicopathological features in NPC.

Variables	No. of patients (n = 65)	INPP4B expression (no. of patients)	
		Absence/Weak (Score = 0–3)	Intermediate/High (Score>3)
**Age (years)**			
≤50	34	17	17
>50	31	15	16
**Gender**			
Male	50	25	25
Female	15	7	8
**Clinical stage**			
Early (stage 1 and 2)	24	9	15
Late (stage 3 and 4)	41	23	18
**Loco-regional recurrence or distant metastasis**			
Absent	45	23	22
Present	20	9	11

### Promoter hypermethylation of *INPP4B* in NPC

**Figure 2:**
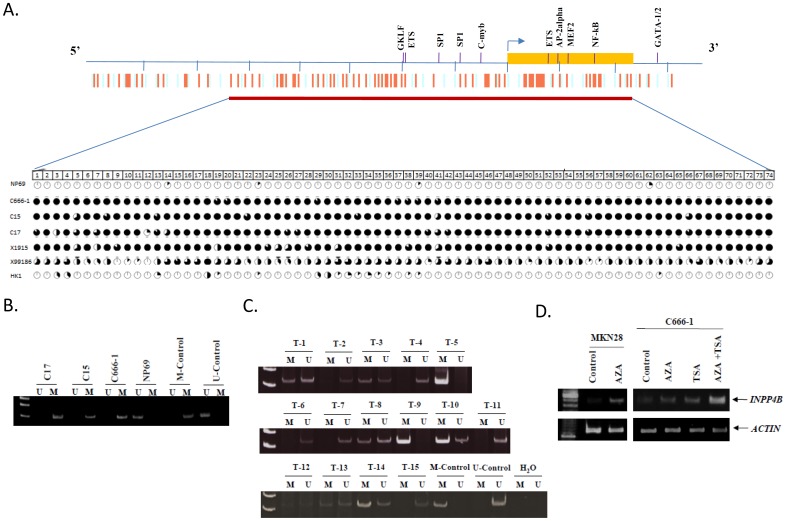
Promoter hypermethylation of *INPP4B* in EBV-positive NPC. (A) Genomic bisufite sequencing of the 5′CpG island of *INPP4B* in NPC cell line and xenografts. Dense methylation was detected in the EBV+ve tumor lines, C666-1, C15, C17, xeno-1915, and xeno-99186. In NP69 (immortalized normal NP cells) and HK-1 (EBV-ve NPC cell line), almost all of the CpG sites were unmethylated. Each circle represents a CpG site and the percentage of methylated alleles is shown as a proportion of the dark area. Eight to ten clones were sequenced for each sample. Yellow box: exon 1; arrow: transcription start site; orange bars: CpG sites. The raw data was available in the MethDB database (http://www.methdb.de/). The link of the data is: http://methdb.univ-perp.fr/cgi/methdbcrossref.cgi?author_id=79. (B) MSP analysis of NPC tumor lines. Methylated alleles were shown in C17, C15 and C666-1. In NP69, only unmethylated alleles were detected. U: unmethylated allele; M: methylated alleles; M-control: methylated control; U-control: unmethylated control. (C) Detection of hypermethylation of *INPP4B* in 8/15 primary tumors (T-1, T-3, T-5, T-8, T-9, T-10, T-12, T-13, T-14). (D) RT-PCR showed the restoration of *INPP4B* expression in the C666-1 cell line after treatment with the DNA methyltransferase inhibitor 5-aza-dC (5-Aza-2′-deoxycytidine) or HDAC inhibitor TSA (Trichostatin-A). Restoration of *INPP4B* gene expression by 5-aza-dC treatment was also shown in the gastric cancer cell line MKN28. The experiments were carried out in triplicate.

Promoter hypermethylation is one of the main mechanisms for the inactivation of cancer-related genes in EBV-associated NPC [13]–[14], [28], [30]–[32]. In the *INPP4B* gene, a 717 bp CpG island that contains 74 CpG sites spanning the promoter region and exon 1 was detected (Figure 2A). We explored the methylation status of this region in the NPC tumor lines by genomic bisulfite sequencing. As shown in Figure 2a, dense methylation of the 5′CpG island was detected in all 5 EBV-positive NPC tumor lines. DNA methylation in this region was rarely detected in the *INPP4B*-expressing NP69 and HK-1 cell lines. A MSP analysis was established for detecting *INPP4B* methylation in both NPC tumor lines and primary tumors (Figure 2B). The hypermethylation of *INPP4B* was detected in 9/15 (60%) of primary NPCs (Figure 2C). To further confirm that the downregulation of *INPP4B* in NPC was due to DNA methylation, we have treated the C666-1 cells with a demethylation agent (5-aza-2′deoxycytidine) or a histone deacetylase (HDAC) inhibitor (trichostatin A). The transcription of *INPP4B* was highly upregulated in the cells treated with both 5-aza-2′deoxycytidine and tricostatin A (Figure 2D). The findings indicate that *INPP4B* transcription in NPC cells is frequently silenced by epigenetic alterations.

### Loss of INPP4B contributes to activated PI3K/AKT signaling in NPC cells

**Figure 3:**
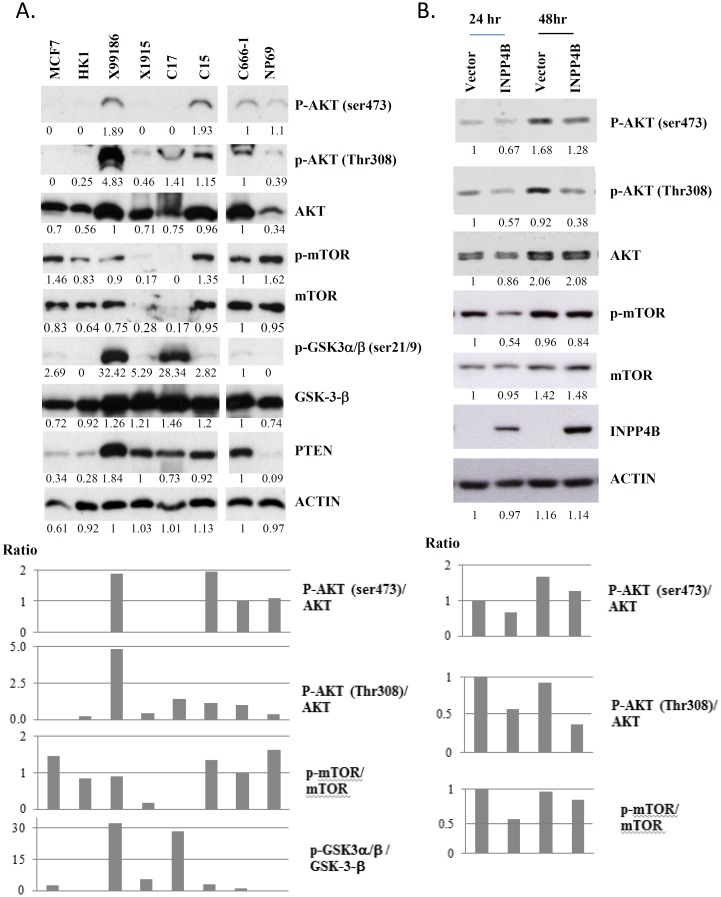
Activation of the PI3K/AKT pathway in EBV+ve NPC tumor cell line and xengrafts. (A) By Western blotting, p-AKT (Thr308) was detected in all EBV-positive NPC tumor lines and p-AKT (Ser473) was found in C666-1, C15 and xeno-99186. The weak expression of p-AKT was shown in the NP69 cells. No AKT activation was observed in the EBV-ve NPC cell line HK-1 and the breast cancer cell line MCF7. p-mTOR was found in all of the cell lines except C17 and xeno-1915 which also shown absence of mTOR expression. The phosphorylation of GSK-3β (Ser9) was detected in xeno-99186 and C17. A high level of PTEN expression was found in the EBV+ve NPC tumor lines. Relative protein expression was calculated using densitometry with C666-1 at 1. The ratios of the phosphorylation and total protein of AKT, mTOR and GSK-3β were also indicated. (B) Suppression of AKT and mTOR phosphorylation was detected in NPC C666-1 cells transient transfected with *INPP4B*. Relative protein expression was calculated using densitometry with vector control (24hr) at 1. The ratios of the phosphorylation and total protein of AKT and mTOR were indicated.

Since INPP4B is a negative regulator of PI3K/AKT signalling, the depletion of INPP4B may result in activation of PI3K/AKT downstream signals [4]–[5]. By Western blotting, we confirmed the increase of p-AKT (Thr308) in the NPC tumor lines (C666-1, C15, C17 and X99186) in which the expression of INPP4B is reduced or absent (Figure 3A). An increase in p-AKT (Ser-473) was also found in C666-1, C15 and X99186. Like INPP4B, PTEN is a negative regulator of the PI3K/AKT pathway. However, a high level of PTEN expression was detected in all 5 EBV-positive NPC tumor lines (Figure 3A). The phosphorylation of mTOR and GSK3-β was also detected in these tumors. The results suggest that *INPP4B* inactivation contributes to the constitutive activation of the PI3K/AKT signaling pathway in these EBV-positive NPC tumors. Ectopic expression of wild type *INPP4B* in the C666-1 cells significantly suppressed the phosphorylation of AKT and mTOR (Figure 3B). This finding confirms that epigenetic inactivation of *INPP4B* plays a role in activation of the PI3K/AKT signaling pathway in NPC cells.

### Restoration of *INPP4B* suppresses *in vivo* tumor growth in NPC cells

**Figure 4:**
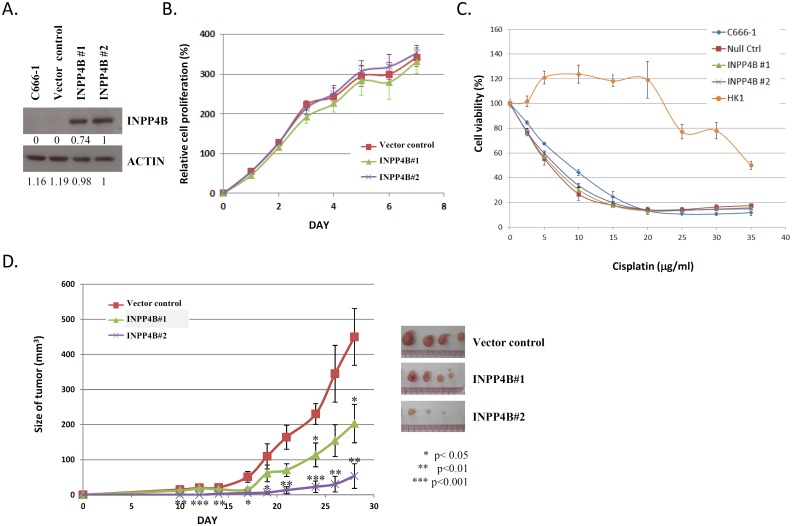
Effect of INPP4B expression on the *in vitro* and *in vivo* growth of NPC cells. (A) The expression of INPP4B was detected in the stable INPP4B-C666-1 clones, INPP4B#1 and INPP4B#2, by Western blotting. Relative protein expression was calculated using densitometry with INPP4B#2 set at 1. (B) No significant inhibition of *in vitro* cell proliferation was detected in the stable INPP4B-expressing C666-1 cells. The WST-1 assay for the detection of the proliferation of INPP4B-expressing and control C666-1 cells was performed in triplicate. (C) Similar sensitivity to the cisplatin treatment was found in the stable INPP4B-expressing C666-1 cells and vector control. The cell viability assays were carried out in triplicate. A cisplatin resistant EBV-negative NPC cell line HK1 was also shown as reference. (D) Significant *in vivo* growth inhibition was observed in the stable INPP4B-expressing cells compared with the control. Four nude mice were injected subcutaneously with stably INPP4B-expressing or control C666-1 cells and the tumor sizes were measured from day 10 to 28 post inoculation. The data are shown as the mean±SEM. A student t-test was used to assess statistical significance, with a p-value of less than 0.05 considered significant (*p<0.05).

To explore whether INPP4B modulate the *in vivo* and *in vitro* growth of NPC cells, two stable INPP4B-expressing C666-1 cell clones, INPP4B#1 and INPP4B#2, were generated (Figure 4A). As shown in Figure 4B, INPP4B expression did not suppress the *in vitro* proliferation of C666-1 cells. The stable INPP4B-expressing cells also demonstrated a similar sensitivity to the cisptain treatment compared with the controls (Figure 4C). Similar finding was also observed in the cisplatin-resistant C666-1 cells transiently transfected with INPP4B (Figure S2). Despite the limited *in vitro* effect on cell proliferation and survival, significant suppression was observed in *in vivo* tumor growth of the cells stably expressing INPP4B (Figure 4D). This finding indicates that the inactivation of *INPP4B* contributes to *in vivo* tumor growth in NPC.

## Discussion

The activation of PI3K/AKT signaling pathway promotes tumor development and resistance to anticancer therapies in human cancers [2]–[3], [11]–[12]. In EBV-associated NPC, activated AKT was demonstrated in 42 to 85% of primary tumors by immunohistochemistry. The phosphorylation of the AKT downstream targets, GSK-3β, FKHR, and BAD was also detected in the majority of cases with activated AKT [2]–[3], [11]–[12]. In this study, we also confirmed the consistent activation of PI3K/AKT signaling in all of the EBV-positive tumor lines examined. Notably, the downregulation of INPP4B was commonly found in both the tumor lines and primary tumors. We proved that the restoration of INPP4B expression inhibited the phosphorylation of AKT and mTOR in the NPC C666-1 cells. Furthermore, the NPC cells stably expressing INPP4B showed reduced *in vivo* tumorigenicity. A similar effect of INPP4B depletion on the PI3K/AKT signaling axis was previously demonstrated in melanoma, breast and prostate cancers [4]–[5], [33]–[35]. In agreement with observations in melanoma, our functional study revealed no obvious effect of INPP4B expression on the proliferation and survival of NPC cells *in vitro* [35]. In a recent study of tumor suppressor function of *INPP4B* in melanoma, Perez-Lorenzo et al. showed that the growth rate in *BRAF/NRAS* wild type melanoma cells on INPP4B knockdown was significantly increased. However, the effect was not observed in melanoma cells with oncogenic *BRAF* or *NRAS* mutations [35]. The finding indicates that the effect of *INPP4B* on cell proliferation is depended on the presence of other genetic changes. Nevertheless, a significant inhibitory effect of INPP4B expression on *in vivo* NPC tumor growth in nude mice was observed, similar to that previously reported in melanoma and breast cancers [5], [33], [35]. The reduced *in vivo* tumorigenicity in the INPP4B-expressing cells strongly supports the tumor suppressor role of *INPP4B* in NPC. Our findings indicate the importance of activating the PI3K/AKT signaling pathway by *INPP4B* depletion in NPC pathogenesis. Up to now, a number of studies have explored the mechanisms that are responsible for activating PI3K/AKT signaling in this EBV-associated epithelial cancer. Studies of EBV-encoded LMP1 and LMP2A have revealed that these latent proteins mediate the transformation of epithelial cells through the activation of PI3K/AKT pathway [17]–[18], [21]. In our earlier study, we also detected the high-level amplification of *PIK3CA* in 20% of primary tumors [22]. In addition to the viral oncoproteins and genetic changes, Zhang also demonstrated that the upregulation of microRNA-144 is able to activate the PI3K/AKT pathway by repressing PTEN expression in NPC cells [36]. Interestingly, we found a high level PTEN expression in the EBV-positive NPC tumor lines which also showed increased phosphorylation of AKT. The expression of PTEN in C666-1 has also been reported in previous studies [36], [37]. In our recent whole genome sequencing study of EBV-positive NPC tumor lines, we did not identify any genetic alterations including indels and mutations of *PTEN* genes (unpublished findings). It is likely that INPP4B is a major target for inactivation in these tumors.

Here, we have shown, for the first time, that *INPP4B* was transcriptionally silenced by promoter hypermethylation. We have demonstrated that the epigenetic inactivation of INPP4B is one of the key mechanisms in activating the PI3K/AKT signaling cascade and thereby contributes to the oncogenesis of EBV-associated NPC.

## Supporting Information

Figure S1 **INPP4B expression and clinicopathological features in NPC patients.** (A) No correlation of INPP4B expression with disease stages was found in the NPC patients. Archival formalin-fixed paraffin-embedded EBV-positive specimens were processed for IHC staining of INPP4B expression. The number and intensity of positive reactions were recorded and correlations were analysed against different clinicopathological features. Archival formalin-fixed paraffin-embedded EBV-positive specimens were processed for IHC staining on INPP4B expression. The number and intensity of positive reactions were recorded and correlations were analysed against different clinicopathological features. (B) INPP4B expression was not associated with recurrence or metastatic diseases. The bar chart shows the number of patients with or without metastasis/recurrence in groups of high/intermediate and low/absent INPP4B expression. Fischer's exact test was used to determine any statistical significance. (C) No significant correlation of INPP4B expression with the overall and disease-free survival of NPC patients was found. The graphs show the survival curves of the NPC patients against INPP4B expression. The survival time of the NPC patients was correlated witht high/intermediate and low/absent INPP4B expression. No significant correlation of INPP4B expression with the overall and disease-free survival of the NPC patients was found. Statistical analysis was performed using the log-rank (Mantel-Cox) test.
(TIF)

Figure S2 **Effect of INPP4B expression on the sensitivity to the cisplatin treatment in the selected cisplatin resistant C666-1 cells.** A cisplatin resistant C666-1 cell line was established and transiently transfected with INPP4B. No significant change of sensitivity was observed in the INPP4B-transfected cells. The cell viability assays were carried out in triplicate.
(TIF)

Table S1 **Primer sequences for RT-PCR, MSP and bisulfite sequencing.**
(DOCX)

Table S2 **Primary Antibodies used in Western blotting.**
(DOCX)
