# Supplementary figures and images for: Epigenetic Inactivation of Inositol polyphosphate 4-phosphatase B (INPP4B), a Regulator of PI3K/AKT Signaling Pathway in EBV-Associated Nasopharyngeal Carcinoma
Source: PLoS One. 2014 Aug 15;9(8):e105163. doi: 10.1371/journal.pone.0105163 (PMC4134277; doi:10.1371/journal.pone.0105163)

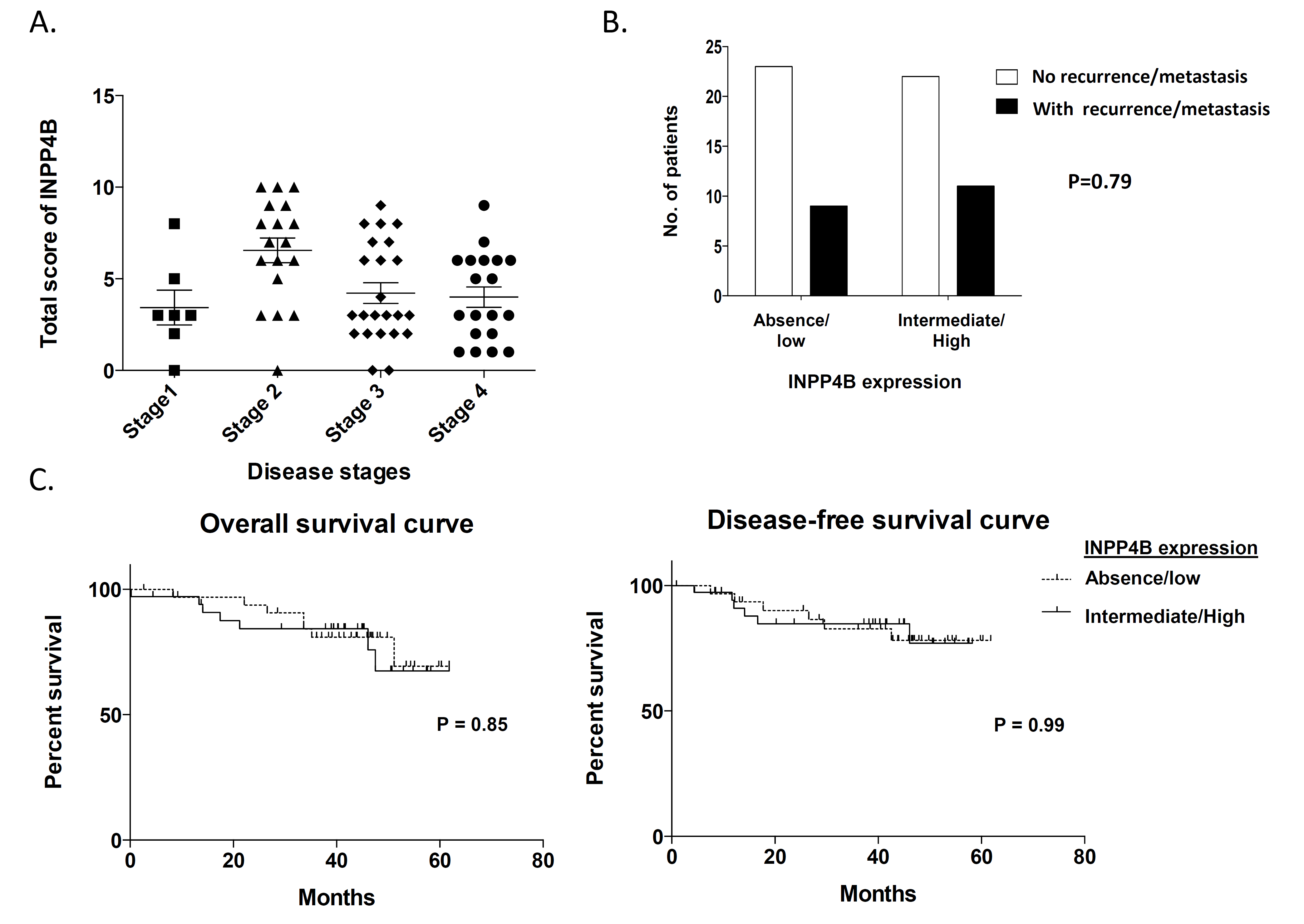

Supplement: Figure S1 — INPP4B expression and clinicopathological features in NPC patients. (A) No correlation of INPP4B expression with disease stages was found in the NPC patients. Archival formalin-fixed paraffin-embedded EBV-positive specimens were processed for IHC staining of INPP4B expression. The number and intensity of positive reactions were recorded and correlations were analysed against different clinicopathological features. Archival formalin-fixed paraffin-embedded EBV-positive specimens were processed for IHC staining on INPP4B expression. The number and intensity of positive reactions were recorded and correlations were analysed against different clinicopathological features. (B) INPP4B expression was not associated with recurrence or metastatic diseases. The bar chart shows the number of patients with or without metastasis/recurrence in groups of high/intermediate and low/absent INPP4B expression. Fischer's exact test was used to determine any statistical significance. (C) No significant correlation of INPP4B expression with the overall and disease-free survival of NPC patients was found. The graphs show the survival curves of the NPC patients against INPP4B expression. The survival time of the NPC patients was correlated witht high/intermediate and low/absent INPP4B expression. No significant correlation of INPP4B expression with the overall and disease-free survival of the NPC patients was found. Statistical analysis was performed using the log-rank (Mantel-Cox) test. (TIF) [file pone.0105163.s001.tif]

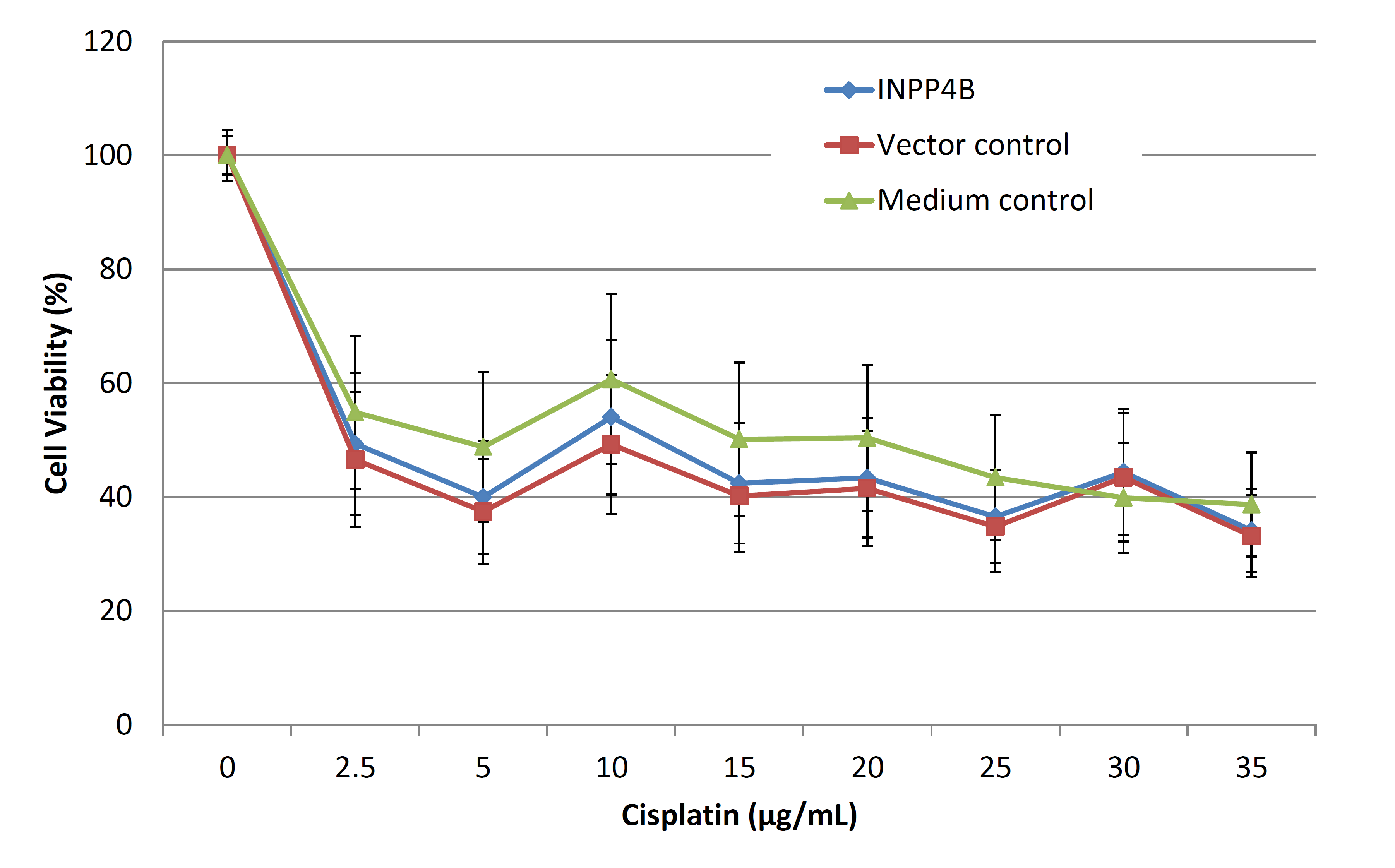

Supplement: Figure S2 — Effect of INPP4B expression on the sensitivity to the cisplatin treatment in the selected cisplatin resistant C666-1 cells. A cisplatin resistant C666-1 cell line was established and transiently transfected with INPP4B. No significant change of sensitivity was observed in the INPP4B-transfected cells. The cell viability assays were carried out in triplicate. (TIF) [file pone.0105163.s002.tif]
