# Supplementary material for: Epigenetic Inactivation of Inositol polyphosphate 4-phosphatase B (INPP4B), a Regulator of PI3K/AKT Signaling Pathway in EBV-Associated Nasopharyngeal Carcinoma
Source: PLoS One. 2014 Aug 15;9(8):e105163. doi: 10.1371/journal.pone.0105163 (PMC4134277; doi:10.1371/journal.pone.0105163)
Supplement: Table S1 — Primer sequences for RT-PCR, MSP and bisulfite sequencing. (DOCX) [file pone.0105163.s003.docx]

Supplementary Table S1: Primer sequences for the RT-PCR, MSP and bisulfite sequencing.

| **RT-PCR:** | | | |
| --- | --- | --- | --- |
|  | Forward (5’ to 3’) | Reverse (5’ to 3’) | Annealing |
| Sequence | GCCGACCACATCACCACAG | TTTCCGCTCACACTTTCCG | 60 ^o^C |
|  | | | |
| **Methylation Specific PCR:** | | | |
|  | Forward (5’ to 3’) | Reverse (5’ to 3’) | Annealing |
| Methylated | TGTTAGGGGGCGTGTGAGTAGTC | CAATACCCGAAACTAAAAACCCGAA | 64 ^o^C |
| Unmethylated | GTGTTAGGGGGTGTGTGAGTAGTTG | CAATACCCAAAACTAAAAACCCAAA | 63 ^o^C |
|  | | | |
| **Bisulfite Sequencing:** | | | |
|  | Forward (5’ to 3’) | Reverse (5’ to 3’) | Annealing |
| Sequence 1 | GAGGGGTAAGAGATAGGGGTTT | CCCACCCAAACTAAACTATAAAATC | 56^o^C |
| Sequence 2 | TTATAGTTTAGTTTGGGTGGG | CCCAAAAAAATTATCACCAAC | 56^o^C |
| Sequence 3 | GTTGGTGATAATTTTTTTGGGG | AACACTTTTAACCTAACTTTCCTAC | 54^o^C |
