# Supplementary material for: Epigenetic Inactivation of Inositol polyphosphate 4-phosphatase B (INPP4B), a Regulator of PI3K/AKT Signaling Pathway in EBV-Associated Nasopharyngeal Carcinoma
Source: PLoS One. 2014 Aug 15;9(8):e105163. doi: 10.1371/journal.pone.0105163 (PMC4134277; doi:10.1371/journal.pone.0105163)
Supplement: Table S2 — Primary Antibodies used in Western blotting. (DOCX) [file pone.0105163.s004.docx]

Supplementary Table S2 : Primary Antibodies used in Western blotting.

| Primary antibody | clone |  |
| --- | --- | --- |
| anti-INPP4B | N-20 | Santa Cruz Biotechnology sc-12318 |
| anti-AKT |  | Cell Signaling #9272 |
| anti-phospho-AKT (ser 473) |  | Cell Signaling #9271 |
| anti-phospho-AKT (ser 308) | 244F9 | Cell Signaling #4056 |
| anti-PTEN | 138G6 | Cell Signaling #9559 |
| anti-mTOR | 7C10 | Cell Signaling #9862 |
| anti-phospho-mTOR(ser2448) |  | Cell Signaling #2971 |
| anti-GSK-3β | 27C10 | Cell Signaling #9315 |
| anti-phospho-GSK-3α/β (ser21/9) |  | Cell Signaling #9331 |
| anti-beta-actin | I-19 | Santa Cruz Biotechnology sc-1616 |
